# Complete Genome Sequence of the Novel Cellulolytic, Anaerobic, Thermophilic Bacterium *Herbivorax saccincola* Type Strain GGR1, Isolated from a Lab Scale Biogas Reactor as Established by Illumina and Nanopore MinION Sequencing

**DOI:** 10.1128/genomeA.01493-17

**Published:** 2018-02-08

**Authors:** Alexander Pechtl, Christian Rückert, Irena Maus, Daniela E. Koeck, Nataliya Trushina, Petra Kornberger, Wolfgang H. Schwarz, Andreas Schlüter, Wolfgang Liebl, Vladimir V. Zverlov

**Affiliations:** a Department of Microbiology, TUM School of Life Sciences Weihenstephan, Technical University of Munich, Freising, Germany; b Center for Biotechnology (CeBiTec), Bielefeld University, Bielefeld, Germany; c Institute of Molecular Genetics, Russian Academy of Science, Moscow, Russia

## Abstract

The cellulolytic bacterium *Herbivorax saccincola* strain GGR1, which represents the type strain of this species, was isolated from the *in vivo* enriched cellulose-binding community of a lab scale thermophilic biogas reactor. Here, we report the complete genome sequence of *H. saccincola* GGR1^T^, the first isolated member of the genus *Herbivorax*.

## GENOME ANNOUNCEMENT

Biogas is a sustainable energy carrier produced in large scale from organic biomass by anaerobic fermentation in biogas reactors. Recent studies have reported on the abundance of cellulolytic *Ruminococcaceae* members within the microbial communities of thermophilic biogas-producing plants (1, 2). Members of this group were isolated from various environments, and some of them are highly efficient lignocellulose degraders that produce cellulosomes (3). The anaerobic bacterium *Herbivorax saccincola* GGR1^T^ was isolated from a thermophilic grass silage/cow manure biogas reactor (4). Phylogenetic analysis based on 16S rRNA gene sequencing classified the new isolate GGR1^T^ as belonging to a hitherto unknown subgroup within the family *Ruminococcaceae*. It was shown to utilize cellulose and xylan as sole carbon sources. Hydrogen, ethanol, and acetate are the major fermentation products (4). To assess its adaptive genome features, the strain GGR1^T^ was completely sequenced using the Illumina and Nanopore MinION sequencing technologies.

For GGR1^T^ genome sequencing, chromosomal DNA was used to generate a shotgun 8-kb mate-pair sequencing library that was sequenced on an Illumina MiSeq system (5). Sequence data were assembled using Newbler version 2.8 (Roche), resulting in 12 scaffolds containing 139 contigs. Subsequently, 2 μg of the GGR1^T^ genomic DNA was used to generate a second shotgun library for sequencing on the MinION system (Oxford Nanopore Technologies). DNA fragments of 5 to 50 kb were used to create a 1D^2^ sequencing library, which was loaded to an R9.5 flowcell for a 24-h run on the MinION sequencer. Base calling and data conversion were performed using Albacore version 1.2.4 (https://github.com/Albacore/albacore). The assembly was performed applying Canu version 1.5 (6). After assembly, the resulting eight contigs were polished with the short Illumina reads using Pilon (7). The final assembly was done manually using Consed (8). This resulted in a circular contig of 3,604,547 bp, featuring a G+C content of 34.82%. Gene prediction and annotation were performed applying Prokka (9) and GenDB (10). This approach resulted in the detection of 3,228 coding sequences, 52 tRNAs, and 3 *rrn* operons.

The GGR1^T^ genome harbors 114 genes encoding carbohydrate-active enzymes identified by means of the carbohydrate-active-enzyme database (CAZy) annotation Web server dbCAN (11). The identified CAZy modules comprise 68 glycoside hydrolases (GHs), 20 carbohydrate esterases, 24 glycosyl transferases, and 2 polysaccharide lyases.

A total of 60 putative cellulosomal genes were identified by the presence of coding regions for type 1 or 2 dockerin or cohesin modules, among others. Fifty of these genes encode 35 different GHs and other carbohydrate-active enzymes. One corresponding primary scaffoldin comprising 11 type 1 cohesin modules and a carbohydrate-binding module (CBM3) was identified in the genome of GGR1^T^ as well as 9 other scaffoldins and anchoring proteins. Two cellulosomal enzymes are unusual because one contains both type 1 dockerin and cohesin modules, and another one likewise harbors type 2 dockerin and cohesin modules. The availability of the *H. saccincola* GGR1^T^ genome sequence provides the genetic basis for biotechnological exploitation of encoded biocatalysts and serves as a reference for the analysis of biomass-digesting microbial communities.

### Accession number(s).

The genome sequence of *H. saccincola* GGR1^T^ has been deposited in the EMBL/GenBank database (EBI, NCBI) under the accession number CP025197. The strain is available from the Leibniz Institute German Collection of Microorganisms and Cell Cultures (DSMZ, Braunschweig, Germany) under strain number DSM 101079.
